# Worthless donations: male deception and female counter play in a nuptial gift-giving spider

**DOI:** 10.1186/1471-2148-11-329

**Published:** 2011-11-14

**Authors:** Maria J Albo, Gudrun Winther, Cristina Tuni, Søren Toft, Trine Bilde

**Affiliations:** 1 Department of Bioscience, Aarhus University, Ny Munkegade 116, Aarhus, Denmark; 2 Laboratorio de Etología, Ecología y Evolución, Instituto de Investigaciones Biológicas Clemente Estable, Avenida Italia 3318, Montevideo, Uruguay

## Abstract

**Background:**

In nuptial gift-giving species, benefits of acquiring a mate may select for male deception by donation of worthless gifts. We investigated the effect of worthless gifts on mating success in the spider *Pisaura mirabilis*. Males usually offer an insect prey wrapped in silk; however, worthless gifts containing inedible items are reported. We tested male mating success in the following experimental groups: protein enriched fly gift (PG), regular fly gift (FG), worthless gift (WG), or no gift (NG).

**Results:**

Males that offered worthless gifts acquired similar mating success as males offering nutritional gifts, while males with no gift experienced reduced mating success. The results suggest that strong selection on the nuptial gift-giving trait facilitates male deception by donation of worthless gifts. Females terminated matings faster when males offered worthless donations; this demonstrate a cost of deception for the males as shorter matings lead to reduced sperm transfer and thus give the deceiving males a disadvantage in sperm competition.

**Conclusion:**

We propose that the gift wrapping trait allows males to exploit female foraging preference by disguising the gift content thus deceiving females into mating without acquiring direct benefits. Female preference for a genuine prey gift combined with control over mating duration, however, counteracts the male deception.

## Background

Differences in the evolutionary interests between the sexes over maximizing reproductive success commonly lead to inter-sexual conflict [1,2]. This conflict influences the opportunity, form and intensity of sexual selection that drives the evolution of traits that enhance mating success [3]. Traits that differ in optimum between males and females may include the number of mates, copulation duration, fertilization success as well as parental investment in offspring [1]. Male-male competition may be a particular strong driver of sexually antagonistic traits; thus to enhance their success in sperm competition, males may be under selection to manipulate females to mate at a suboptimal rate [2,4].

In species where males provide females with a nuptial gift during mating, there is particular scope for males to manipulate females to acquire matings and prolong copulation to enhance their fertilization success [4-6]. Female choice for males with nuptial gifts could lead to the evolution of male "deception" by the use of token gifts. For instance, males can decrease the costs of mating by re-using gifts or by offering worthless gifts [7,8]. Males of some dance flies (*Empis *spp.) may deceive females by offering inadequate or false gifts [8]. Although males that offer inedible gifts run a higher risk of being rejected and may suffer from shorter matings compared to males offering edible gifts, the chance of acquiring an extra mating should make deception an attractive strategy for males. Hence, males of the dance fly *Rhamphomyia sulcata *that use inedible token gifts to obtain mates are as successful as males offering small genuine gifts [9].

In spiders, nuptial gifts in the form of prey are restricted to a few species from two families belonging to the superfamily Lycosoidea: Pisauridae and Trechaleidae [10-17]. In both families, the male courts the female by offering a prey wrapped in silk and mating occurs while the female consumes the gift [15,18]. In the species *Pisaura mirabilis *(Pisauridae) the gift functions as a mating effort that increases male mating success [19]; a similar function was recently suggested for the trechaleid spider, *Paratrechalea ornata *[20]. In both species, males can obtain mating without a gift, but male mating success increases dramatically when a gift is offered [19,20]. In *P. mirabilis*, the male pushes up the female during mating and performs alternate pedipalp insertions into the female sperm storage organs placed ventrally on the abdomen. After each insertion the male returns to a face-to-face position with the female, grabbing the gift in the chelicerae [21]. Females usually control mating duration and they often attempt to run away with the gift upon terminating the copulation [19]. During each insertion the male and the female remain motionless. However, if the female moves and attempts to terminate copulation, the male may perform "thanatosis" which is a "death feigning" behaviour (with stretched-out legs) unique to this species. The male ends the insertion and grasp the gift with his chelicerae. The female moves away while holding the nuptial gift and the male is dragged along until the female stops. Subsequently he "revives" and resumes mating [22-24]. Thanatosis functions as a mating effort that increases the male's chances of completing or prolonging the copulation [23]. Silk wrapping facilitates male handling and control over the gift [25], as it facilitates a stronger hold of the silk covered package, both with the chelicerae and the feet claws, compared to an unwrapped insect. As a result, females are less likely to succeed in stealing a wrapped than an unwrapped gift [26,27]. In addition, Stålhandske [28] showed that relatively brighter gifts, i.e. gifts that were wrapped in plenty of white silk, were more attractive to females, suggesting an additional function of silk wrapping that directly influences mate choice. The prey gift and silk wrapping thus provide males with opportunities to exploit female foraging preferences in a sexual context [29].

Male spiders have a unique opportunity for gift manipulation through the gift wrapping trait, for example by preventing female assessment of the gift content. By disguising the gift content males may deceive females to copulate, while the female attempts to consume the gift. In *P. ornata*, males were observed wrapping prey carrion and occasionally inedible items such as plant seeds [20]. In *P. mirabilis*, males have been reported to carry gifts containing empty arthropod exoskeletons or plant parts [21,30,31], gifts that are of no nutritional value. Dissection of 16 gifts carried by males in the field showed that 62% contained fresh prey, while the remaining 38% contained empty arthropod exoskeletons, i.e. prey already sucked out probably by the male itself [M.J. Albo, unpublished data]. Such evidence suggests that males of *P. mirabilis *may exploit the female's preference for prey gifts to gain a reproductive advantage without providing the nutritional benefit of fresh prey from which the gift-giving trait must ultimately have evolved [29]. The costs of prey capture and gift construction may thus interact with male condition to favour the evolution of male deceit by donation of worthless gifts [32].

Here we tested male use of worthless gifts and investigated their effect on male and female reproductive success in a Scandinavian population of *P. mirabilis*. We explored how the gift content affects reproductive success in experimental trials where males offered either genuine prey gifts, worthless gifts, or no gift to females. Males are under strong selection to provide a gift [19]; therefore, we expected males to offer a worthless (non-nutritive) item if no prey is available. Males offering worthless gifts may initially be accepted by females, however during feeding the females should realize the low nutritional value of the gift and respond appropriately, e.g. by interrupting the mating prematurely. As a consequence, males would experience shorter matings, lower sperm transfer and ultimately lower reproductive success than males offering genuine gifts.

## Results

### Courtship and mating

**Figure 1:**
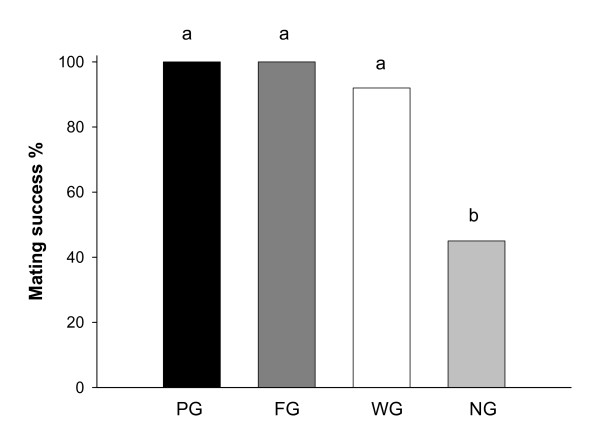
**Mating success of male *P. mirabilis *spiders offering different nuptial gifts, PG (protein gift), FG (fly gift), WG (worthless gift) and NG (no gift)**. Different letters indicate significant differences from pair-wise Chi-square tests.

**Figure 2:**
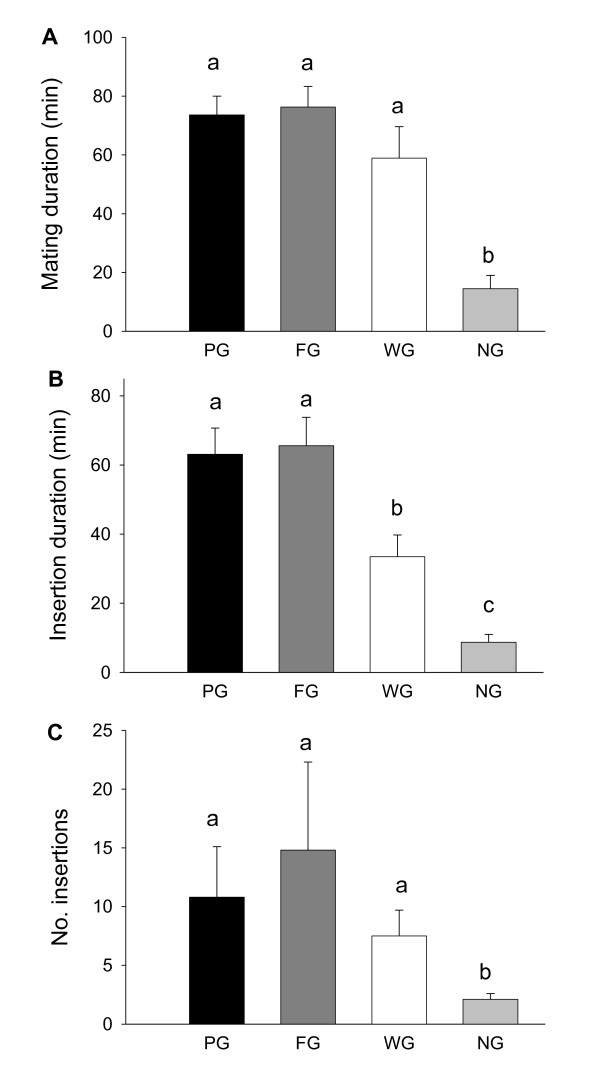
**Mating behaviours of male *P. mirabilis *spiders offering different nuptial gifts (PG (protein fly gift), FG (fly gift), WG (worthless gift) and NG (no gift), data presented as mean and standard error)**. A) Mating duration, B) insertion duration and C) number of insertions. Statistical comparisons were performed using two-way ANOVA or Kruskal-Wallis test, and the pair-wise comparisons using Student t-test or Mann-Whitney U test, respectively. Groups with the same letter were not significantly different (p > 0.05).

In the worthless gift group (WG), 13 males (70%) produced a worthless gift for courtship and 12 out of these mated, whereas the remaining 6 males (30%) courted without a gift and only one mated (Fisher exact test: p = 0.002). Comparing all treatment groups, males that offered a worthless gift (WG) were equally successful in obtaining mating as those offering a genuine gift (PG and FG), whereas males with no gift (NG) experienced significantly reduced mating success (Chi-Square test: χ^2^_yates _= 24.8, p < 0.0001, df = 3; Figure 1). The total mating duration was significantly shorter for NG males compared with males that offered a gift (F = 10.03, p < 0.0001, df = 3, Figure 2A). Although WG males experienced a 20% shorter mating duration compared with PG and FG males, this effect was not statistically significant (Figure 2A). Insertion duration, which reflects the duration of actual sperm transfer, was significantly shorter for WG males compared with males offering genuine gifts, and shortest for males with no gift (H = 41.32, p < 0.0001, df = 3, Figure 2B). The number of pedipalp insertions followed a similar pattern (H = 7.8, p = 0.05, df = 3, Figure 2C).

### Male thanatosis and gift control

Male death feigning (thanatosis) occurred in more than 50% of the trials in the PG and FG groups, with only one occurrence in the WG group and none in the NG group (Table 1). In the PG and FG groups, females terminated mating and retained the gift in all mating trials, in 11 out of 40 cases females and males were observed fighting over the gift. In the WG group, all females that accepted the non-nutritive gift actively manipulated it, moving the item with the chelicerae and pedipalps in the same way as females from the PG and FG groups. Cotton balls were wet after handling, suggesting that the females had regurgitated digestive fluids on them in an attempt to feed on the gift. We did not observe fights between males and females over the non-nutritive items.

**Table 1:** Frequencies of male thanatosis and female gift control in PG (protein fly gift), FG (fly gift), WG (worthless gift) and NG (no gift) groups.

	PG (n = 20)	FG (n = 20)	WG (n = 12)	NG (n = 9)	Statistics		
					χ^2^	P	DF
**Male thanatosis**	12 a	11 a	1 b	0	12.1	= 0.006	3
**Female gift control**	20 a	20 a	1 b	-	40.0	< 0.00001	2

### Worthless items and gift construction

**Figure 3:**
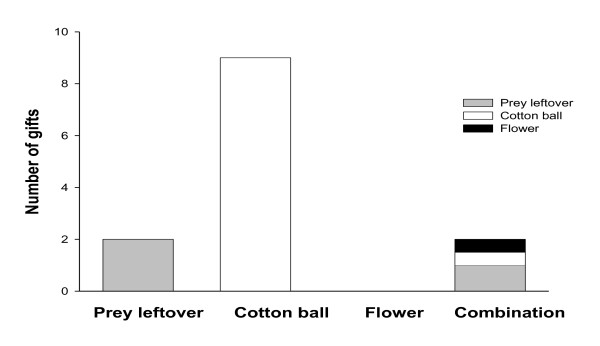
**Occurrences of the three types of worthless items (or combination of items) offered to *P. mirabilis *females**. Males wrapped and offered: a prey leftover, a cotton ball, or a dried out flower head. Two males wrapped two of these items together (combination: a prey leftover plus flower head; and prey leftover plus cotton ball).

In the WG group, the males preferred cotton balls over prey leftovers and flower heads (Chi-Square test: χ^2^_yates _= 14.8, p = 0.001, df = 3, Figure 3). Two males wrapped and combined two items: one male combined a prey leftover and a flower head, while the other combined a prey leftover and a cotton ball. To measure male silk investment in worthless gifts, we compared the duration of gift wrapping for WG and FG gifts. Males with worthless gifts spent less time on gift construction (mean ± SE, 3.9 ± 0.5 min, N = 12) than those with fly gifts (5.3 ± 0.5 min, N = 20) (Student t-test: t = 2.04, p = 0.049) and showed a tendency to perform fewer wrapping bouts (3.0 ± 0.4) than males with fly gifts (4.1 ± 0.3) (Student t-test: t = 1.80, p = 0.08). Males with cotton ball gifts (preferred item by WG males) performed a significantly lower number of wrapping bouts (2.5 ± 0.3, N = 8) compared to males with fly gifts (4.1 ± 0.3, N = 20) (Mann-Whitney U-test: U = 37, p = 0.03).

### Oviposition, spiderling emergence and female life span

We found no significant differences in the proportion of mated females that constructed an egg-sac among groups; 19 (20) PG females, 20 (20) FG females, 12 (12) WG females and 8 (9) NG females constructed an egg-sac (Chi-Square test: χ^2^_yates _= 0.20, p = 0.97, df = 3). The time until egg-sac hatching was similar among groups: 18.1 days (± 0.4 SE) in PG, 18.9 days (± 0.8 SE) in FG, 17.0 days (± 1.2 SE) in WG and 17.5 days (± 0.5 SE) in NG (ANOVA: F = 0.85, p = 0.48, df = 3). Egg hatching success differed among groups, and was higher when females had received a gift (PG, FG and WG) compared with no gift (Table 2). We found no significant differences in clutch-size or the number of spiderlings that emerged from the egg-sac among groups (Table 2). Spiderling size differed among groups, the largest spiderlings appeared to be found in the WG group, however a significant treatment by clutch-size interaction and a significant positive co-variation between clutch size and spiderling size make a simple interpretation of these data difficult (Table 2).

**Table 2:** Female fitness traits across nuptial gift mating treatments, PG (protein gift), FG (fly gift), WG (worthless gift) and NG (no gift), data are presented as means and standard error.

	PG	FG	WG	NG	Statistics		
						P	DF
**% Egg hatching success**	0.66 ± 0.10 a (n_es _= 12)	0.50 ± 0.15 a (n_es _= 11)	0.48 ± 0.13 a (n_es _= 8)	0.33 ± 0.33 b (n_es _= 3)	χ^2^_yates _= 91.4	<0.0001	3
**Clutch-size**	45.5 ± 4.5 a (n_es _= 12)	38.0 ± 4.7 a (n_es _= 11)	43.8 ± 5.5 a (n_es _= 8)	27.3 ± 9.0 a (n_es _= 3)	F = 1.30	0.29	3
**Number of spiderlings per egg-sac**	31.7 ± 6.1 a (n_es _= 12)	18.2 ± 6.5 a (n_es _= 11)	23.7 ± 7.6 a (n_es _= 8)	6.3 ± 12.3 a (n_es _= 3)	F = 1.45	0.24	3
**Size of spiderlings (mm+SE)**	0.649 ± 0.003 a (n_sp _= 211; n_es _= 12)	0.672 ± 0.004 b (n_sp _= 107; n_es _= 7)	0.674 ± 0.004 b (n_sp _= 103; n_es _= 7)	n/a	χ^2^_overall _= 86.92 χ^2^_group_= 56.11 χ^2^_clutch-size _= 52.49 χ^2^_group*clutch-size_= 27.56	<0.0001 <0.0001 <0.0001 <0.0001	5 2 1 2

n_sp _= spiderlings sample size; n_es_= egg-sac sample size.

Spiderling size was analyzed with GLM with clutch size as covariate. Groups with the same letter were not significantly different (p > 0.05).

Mated females did not differ in adult lifespan among groups (ANOVA: F = 0.93, p = 0.42, df = 3; PG females 102.9 (± 10.6 SE) days, FG females 91.6 (± 6.4 SE) days, WG females 113.9 (± 10.0 SE) days, and NG females 107.9 (± 14.4 SE) days, N = 61).

## Discussion

We showed that *P. mirabilis *males that offered worthless gifts acquired similar copulation success as males offering genuine nutritional gifts. In contrast, males with no gift experienced a significantly reduced mating success. Previous studies have demonstrated strong female preference for a prey gift (genuine gift), suggesting direct benefits to females of receiving a nuptial gift [29]. Together these findings support the hypothesis that strong selection on the nuptial gift-giving trait facilitates the evolution of male deception through donation of worthless gifts. We suggest that the gift wrapping trait allows males to take advantage of the female preference for a gift by disguising the gift content, thus deceiving females into mating without acquiring a direct benefit. Probably gift wrapping was crucial for deceit to evolve, while gift wrapping itself evolved due to additional advantages to the male: it increases male control over the gift and mating [25,27], and mating duration increases with the amount of silk invested in wrapping [26].

Male deceit may be costly for females if it results in a higher than optimal mating rate, without conferring females a direct benefit [2,4]. If deception with worthless gifts is common [[21,30,31], M.J. Albo personal observations], females should evolve the ability to discriminate nutritive and non-nutritive gifts and avoid mating with males offering worthless gifts. Indeed we found that females terminated copulations with males that offered worthless donations sooner than copulations with males offering genuine gifts. This supports the hypothesis that the female can not evaluate the gift's value before having fed on it for some time. Males with worthless donations experienced shorter insertion duration and thus had less time to transfer sperm. This reveals a cost of deception for males, as shorter copulations and reduced sperm transfer disadvantages males in sperm competition [33]. Females that received a worthless gift did not retain the gift post-mating while they always retained genuine prey gifts, indicating that the nutritional value of the gift was actually revealed to the female during gift consumption and mating. Female preference for a genuine prey gift combined with her control over mating duration therefore counteracts male deception. However, males appear to hold the upper hand in the co-evolutionary cycle, since deception is only discovered after the copulation is initiated.

Males offering worthless gifts were less likely to feign dead (thanatosis), which is puzzling, as thanatosis functions as a male mating effort [22,23]. However, males often feign death if the female attempts to steal the gift without mating [23]. As the females did not try to run away with the worthless gifts, there was less scope for males to use thanatosis to prolong the copulation.

We did not detect strong negative effects of worthless donations on female reproductive fitness. Females receiving no gift, however, experienced reduced egg hatching success. This may suggest that successful sperm transfer (and hence egg hatching) is tightly coupled to whether the female receives a gift or not, but not to actual gift content (genuine or worthless). Males that mated without a gift experienced shorter copulations and fewer pedipalp insertions, indeed indicating that the gift facilitates sperm transfer, at least through its effect on mating duration. This produces a strong incentive on males to present any type of gift rather than no gift. While copulation is possible without a gift, the short mating and insertion durations make mating without a gift unsuccessful. To some extent this also holds for mating with worthless gifts. We found an effect of gift type on spiderling size, though not in the direction expected if worthless gifts had negative effects on spiderling size. Instead, females receiving protein gifts which were presumably of the highest nutritional quality produced the smallest spiderlings; this effect was partly explained by the positive co-variation between clutch size and spiderlings size. Positive nutrient effects of the gift might be revealed in female adult life span [34,35], however, female longevity was similar among groups. Overall we found no indication of a cost of worthless donations on female reproductive fitness.

As females in our study received only a single gift, it is possible that the small amount of nutrients obtained through one gift is insufficient to detect an effect of nuptial gift quality on female fitness traits. *Pisaura mirabilis *females are polyandrous [36,37], and may thus benefit from receiving multiple nuptial gifts. Nuptial feeding may be particularly beneficial for females in poor feeding condition, and such females would benefit from the ability to choose males with a nutritive gift [7,38]. The cost for females of receiving worthless gifts is expected to vary with natural mating rates and to be high under poor foraging conditions.

Among the non-nutritive items, males showed preference for using the cotton ball rather than the other inedible items as a nuptial gift. Since the cotton ball visually appears to resemble a silk wrapped gift, it is possible that males took advantage of this characteristic. The preference may be for both colour (white) and shape (round): Stålhandske [28] showed increased female preference the whiter the gift, and Andersen et al. [27] showed male preference for a round gift compared with an elongate one, probably because the latter mechanically inhibited his access to the female's genitalia. These features suggest that nuptial gift-giving spiders may be more restricted in the availability and suitability of worthless items to use as gifts compared to, for example, the empidid dance flies (9). Empidid dance flies offer a variety of token gifts that are not disguised, for example seed tufts, leaves, or small twigs, these gifts should be less costly to acquire than those of spiders, as they do not require prey capture and gift construction [7-9]. In contrast, spider males are more conservative, they wrap insect exoskeletons which could be prey remains and only occasionally use plant material to construct gifts. Hence, even worthless donations are costly for the male to produce as they require investment in silk and time for gift production. Indeed gift production was shown to be costly, as *P. mirabilis *males in poor condition are constrained in their ability to construct nuptial gifts [32].

## Conclusions

There are large benefits to males of achieving a mating with a worthless donation, favouring male deception and the evolution of worthless gifts. However, the potential disadvantage in sperm competition through shorter copulations effected by the females coupled with costs of gift construction counteracts deceit. This may explain the maintenance of both mating tactics in the population and the prevalence of matings with genuine gifts.

## Methods

We collected *Pisaura mirabilis *(Pisauridae) juveniles and subadults in April 2009 on grasslands surrounding the Mols Laboratory close to Aarhus, eastern Jutland, Denmark. In the laboratory, spiders were housed individually in vials (30 ml) containing water and moss (*Sphagnum *spp.) for maintaining humidity. The spiders were kept at an average room temperature of 23.1°C (± 0.3 SE) and a natural photoperiod. We raised spiders until adulthood, registering the day of each moult to maturity. Individuals were fed three times per week with houseflies (*Musca domestica*). Mating experiments were conducted from 7 to 27 May, 2009. Observations were made in transparent plastic terraria (22 × 17 × 6 cm), with the bottom covered with paper and containing a dish (5 cm diameter) with wet cotton wool. All individuals used during the mating trials were virgins; males were of an average adult age of 19.5 (± 0.6 SE) days and females 19.9 (± 0.6 SE) days. Individuals were used only once.

To investigate the use and reproductive consequences of worthless donations in *P. mirabilis *we carried out experiments where females were exposed to males offering one of three gift types, or no gift: PG (protein fly gift), FG (normal fly gift), WG (worthless gifts), NG (no gift) (20 trials for each group). Protein enriched flies were chosen to examine the effect of gift nutrient quality on female reproductive success [39]. We assumed the following order of gift quality: PG> FG > WG = NG for females ranked according to nutritional value, and PG ≥ FG > WG > NG for males in terms of cost of gift production. Normal flies (*Musca domestica*) were raised on standard housefly medium, whereas protein flies were *Musca domestica *obtained by raising the larvae in a medium consisting of 40% standard house fly medium and 60% casein. Since PG flies were smaller than FG flies (mean ± SD weight, 70.8 ± 2.2 mg and 129.2 ± 3.8 mg, respectively), we supplied PG males with two PG flies to provide approximately the same mass of prey. Worthless gifts consisted either of a cotton wool ball, a dry flower head (*Malus *sp.), or a prey leftover (housefly) previously eaten by a female. The three WG items were of approximately similar size compared to a normal housefly (FG). Flower heads and prey leftovers have been reported as wrapped gifts in gift-giving spider species [[21,30,31], M.J. Albo unpublished data] whereas the cotton wool ball was chosen following the protocol of LeBas & Hockham [9] and because of its visual similarity to the natural gift wrapped in silk.

Males and females were randomly assigned to treatments. For each trial a female was placed in the experimental terrarium one hour before initiating the mating experiment, allowing her to release draglines, which are important stimuli for the male in mate search, and for inducing courtship behaviour and gift construction [30]. Immediately before the trials, we removed the female from the terrarium and allow the male contact with female silk. In the FG and PG groups, once the male initiated courtship we offered the prey with forceps, and the male grasped it and wrapped it in silk. No gift was provided in the NG group. In the WG group, we sequentially and in random order offered one of each worthless gift type to each male (a flower head, a prey leftover, and a small cotton ball); males could chose to wrap one or more items in the same silk package ("combination"). This procedure allowed us to see which non-nutritive gift type was preferred by males. If the male did not grasp and/or wrap any of these items within 15 min, we provided a live housefly to assure that the male was able to wrap. Males that did not accept and wrap this fly were discarded; those that accepted and wrapped had the fly removed and were re-exposed to the same non-nutritive items (this time dispersed on the floor of the terrarium). Therefore, in the WG group some females were exposed to males carrying a gift and others not carrying one. Males without an item could grasp one after contact with the female, or they could copulate without offering a gift. The latter were excluded from comparative analysis; hence only males from the WG group that obtained a mating with a worthless gift were included in comparative analysis across treatment groups. The experiments started by returning the female to the terrarium and were terminated 10 min after the end of copulation or after 30 min if no interactions between the male and the female occurred.

Gift wrapping may occur in separate bouts of adding silk [31] both before and after the male encounters a female. To estimate differences in male investment in gift production between worthless gifts (WG) and normal gifts (FG), we measured the duration of gift construction and counted the number of gift wrapping bouts. Courtship duration (in min) was measured from when the males initiated courtship until copulation was initiated. We registered the occurrence of male thanatosis, i.e. males "feigning death" during courtship and mating [22] and the occurrence of females retaining the gift after mating ("female gift control"). Mating duration was measured from the beginning of the first to the end of the last pedipalp insertion and included the time the male and female were in face-to-face position and handled the gift. Insertion duration was measured from pedipalp insertion until the pedipalp disengagement, and the sum of all insertion durations was considered the total duration of sperm transfer. Courtship and mating data from the WG group included only males that offered an item.

Mated females were subsequently kept individually in the same vials they were raised in and continued with the same feeding regime. This took place at room temperature; however light bulbs were placed 20 cm above the vials raising the temperature to 25.0 °C (± 0.2 SE) during 3 hours at noon, to enhance the hatching success of the egg-sacs. We registered the latency of egg-sac hatching (period from egg-sac construction to the emergence of spiderlings), clutch-size of the first egg-sac (spiderlings + unhatched eggs), egg hatching success, and the number and size of spiderlings among experimental groups. Cephalothorax (prosoma) width of 20 randomly selected spiderlings from each egg-sac was measured under the stereo microscope, to evaluate the effect of gift type on offspring size. Adult female life span was compared between mated females of each group.

Statistical analyses were performed using JMP 7.0 software (SAS institute) and Past [40]. Assumptions of parametric tests were examined using Shapiro-Wilk tests for normal distribution of residuals, and Levene's test for homogeneity of variances. Data were log or sqrt transformed whenever necessary to meet parametric assumptions. For continuous data, we performed ANOVA using Student t-test for planned pair wise comparisons. Non-parametric tests (Kruskal-Wallis and Mann-Whitney) were applied to ordinal data and when assumptions for parametric tests were not met. Spiderling size was analysed by ANCOVA (GLM modelling) with the number of eggs in the egg sac (clutch-size) as covariate. Frequencies were analysed with Chi-square tests (with Yates correction) or Fisher's exact probability test. All tests were two-tailed.

## Authors' contributions

MJA, CT, ST and TB contributed with the conceptual development of the work and the writing of the manuscript. MJA and GW carried out the experiments; MJA and TB performed data analyses. All authors read and approved the final version of the manuscript.
